# Associations of GP practice characteristics with the rate of ambulatory care sensitive conditions in people living with dementia in England: an ecological analysis of routine data

**DOI:** 10.1186/s12913-021-06634-7

**Published:** 2021-06-29

**Authors:** Emily Eyles, Maria Theresa Redaniel, Sarah Purdy, Kate Tilling, Yoav Ben-Shlomo

**Affiliations:** 1 The National Institute for Health Research and Applied Research Collaboration West (NIHR ARC West), University Hospitals Bristol and Weston NHS Foundation Trust, 9th Floor, Whitefriars, Lewins Mead, Bristol, BS1 2NT UK; 2 Population Health Sciences, Bristol Medical School, University of Bristol, Canynge Hall, 58 Whiteladies Rd, Bristol, BS8 2PL UK

**Keywords:** ACSC, Dementia, Admissions, Ambulatory care, Emergency admissions

## Abstract

**Background:**

Hospital admissions for Ambulatory Care Sensitive Conditions (ACSCs) are potentially avoidable. Dementia is one of the leading chronic conditions in terms of variability in ACSC admissions by general practice, as well as accounting for around a third of UK emergency admissions.

**Methods:**

Using Bayesian multilevel linear regression models, we examined the ecological association of organizational characteristics of general practices (ACSC n=7076, non-ACSC n=7046 units) and Clinical Commissioning Groups (CCG n=212 units) in relation to ACSC and non-ACSC admissions for people with dementia in England.

**Results:**

The rate of hospital admissions are variable between GP practices, with deprivation and being admitted from home as risk factors for admission for ACSC and non-ACSC admissions. The budget allocated by the CCG to mental health shows diverging effects for ACSC versus non-ACSC admissions, so it is likely there is some geographic variation.

**Conclusions:**

A variety of factors that could explain avoidable admissions for PWD at the practice level were examined; most were equally predictive for avoidable and non-avoidable admissions. However, a high amount of variation found at the practice level, in conjunction with the diverging effects of the CCG mental health budget, implies that guidance may be applied inconsistently, or local services may have differences in referral criteria. This indicates there is potential scope for improvement.

**Supplementary Information:**

The online version contains supplementary material available at (10.1186/s12913-021-06634-7).

## Background

Dementia affects 448,348 people (4.3 per 100 population) over 65 in England [1] and approximately 40,000 people under 65 are living with dementia (3.4 per 10,000 population) [1, 2]. NICE estimates that a further 200,000 people over 65 are undiagnosed [3]. Dementia costs the UK economy £23 billion per year, more than heart disease, cancer, or stroke, and this is set to rise as the population ages [2, 4]. By 2040, the prevalence of dementia is estimated to double, and the associated costs to triple [2]. Dementia is a priority area in the NHS Five Year Forward View [5], as well as the NHS Long Term Plan [6]. People with dementia (PWD) often have more complex health needs than in the general population [7], and potentially avoidable admissions such as falls, fractures, and infections are more common amongst them than those without dementia [8–10].According to Wolf et al. [11], potentially avoidable admissions account for 9% more hospital episodes in PWD than those without. In England, unplanned hospital admissions account for 67% of hospital bed days, costing £12.5 billion annually [12]. It is estimated that 25% of hospital beds are occupied by PWD[13], and they are admitted more frequently than those without [14]. Between 2008 and 2013, emergency admissions for PWD increased by 48% to nearly 300,000 [4]. Length of stay [13, 15] and mortality [8] are higher than that for non-dementia patients having the same diagnosis and are more likely to result in care home placement rather than independent living [16]. PWD are also more likely to be re-admitted [15, 17]. Dementia is one of the leading chronic conditions in terms of variability in hospital admissions from general practices, even after controlling for demographic factors such as age and sex [12].

Emergency admissions for Ambulatory Care Sensitive Conditions (ACSCs) are potentially preventable given appropriate management in primary or social care [18]. The premise of ACSCs is that effective treatment of acute conditions, good management of chronic illnesses, and immunisation against infectious diseases can reduce the risk of a specified set of hospitalisations [19, 20]. Further, acute conditions which could require an admission can often be managed effectively in the community if identified and treated early including rapid response community care or ambulatory care in a hospital; emergency department or observation unit [21]. Such treatment does not “count” as an inpatient admission and reflects local provision of services to reduce admission avoidance at the primary/secondary care interface [21]. The seven conditions which account for 75% of all hospital spells classified as ACSCs in the NHS are: asthma, diabetes complications, ear, nose and throat infections, convulsions and epilepsy, cellulitis, chronic obstructive pulmonary disease, and influenza and pneumonia [22]. The NHS describes ACSCs in its Directory of Ambulatory Emergency Care for Adults, citing ambulatory care as an important component of improving patient outcomes and experience, and reducing costs, hospitalisations, and length of stay [21]. Purdy et al. [18] developed the classification of ACSCs used in this paper, validating them through the literature and further in Discussion with expert clinicians, policymakers, and researchers in England. This approach was also employed by Sundmacher et al. [19], using group consensus methods to combine existing evidence.

Various factors affect unplanned ACSC admission rates including living in high deprivation areas, poor continuity of care, secondary care bed availability, increased rurality, and non-white ethnicity [14, 22]. There are some studies on the patient level factors associated with ACSC admissions in PWD [11, 17, 23] Furthermore, Busby et al. [20] found considerable differences in general population ACSC admission rates between English GP practices. Phelan et al. [24] found significantly higher ACSC admission rates for PWD compared to those without. However, there is a dearth of studies specifically focused on how general practice related factors are associated with ACSC admissions in PWD [10, 12, 25], despite primary care, and access to it, being a major element in reducing ACSC admissions in general [26].

This study aims to explore health care organisational risk factors for emergency hospital admissions for ACSCs in PWD in England. The National Health Service (NHS) is the main provider of healthcare in England, predominantly through general practitioner (GP) surgeries in primary care, and through hospital trusts in secondary care. These are overseen by Clinical Commissioning Groups (CCGs), which are “clinically-led statutory NHS bodies responsible for the planning of health care services for their local area” [27]. We hypothesised that practices with more experience of dementia care, i.e. an older patient population and more dementia patients, better access and quality and locally larger budgets for mental health care would have lower rates of all admission. We argued a priori, that if any of these factors were causally related to reduced admission rates we would observe a larger effect for ACSC compared to non-ACSC admissions.

## Methods

### Data sources

The primary data source for this study was the Hospital Episode Statistics (HES) database [28], which contains details of admissions in secondary care hospitals in England. NHS Providers and NHS Digital carry out validity and other data quality checks prior to data release. Information collected includes: primary diagnosis, admission source (whether patient was admitted from home or not), patient region of residence, rurality (dichotomised as rural or urban), and the year the admission took place [28]. Other data sources used include the GP Practice Profiles [29], GP Patient Survey [30], the Quality Outcomes Framework (QOF) [31], and the CCG Programme budgeting benchmarking tool [32], as described in Table 1).

**Table 1:** Data sources and variables

Data Source	Description	Variables and variable description
Hospital Episode Statistics (HES)[28]	Information that is required to be collected by secondary care providers	∙ Hospital admissions of people with dementia
		∙ Admission source – where most patients were admitted from (from home or not, reference: not from home). Not from home is a combined category, including all other sources, such as other NHS providers, local authorities, or penal establishments.
		∙ Region of England of practice, of which there are 9 (reference: London)
		∙ Rurality, i.e. whether a practice is located in a rural or urban area, dichotomised from the rural-urban index (reference: urban) Year (the year the admission took place, reference: 2017)
GP Practice Profiles [29]	Collates data that GPs report to the NHS. The data here refer to all patients in a practice, not just people with dementia	∙ Mean practice age
		∙ Percent of female patients
		∙ Index of multiple deprivation (IMD), a measure of relative deprivation of the practice’s area, with a higher score indicating more deprivation.
		∙ Open/close date of practices
		∙ Archived practices (those closed prior to the beginning of the previous financial year)
		∙ The patient population of each practice, split into quartiles, Reference category: the largest practice size quartile
GP Patient Survey [30]	Records patient experiences in GP practices	∙ Percentage of practice access rated as “good” or not by patients
Quality Outcomes Framework (QOF) [31]	Quality measures for GP practices based on several indicators, covering management of chronic conditions and public health concerns, as well as preventative service provision, agreed on by contract negotiations each year.	∙ Number of patients in the practice with dementia
		∙ QOF score – a measure of whether or not a practice meets quality criteria, here dichotomised as ≤90% achievement and >90% achievement. Each indicator adds points to the total. (reference: ≤90%)
CCG Programme budgeting benchmarking tool [32]	Performance statistics collected by CCGs to support evidence-based commissioning of services	∙ Percentage mental health spend for CCGs

### Study population

Our Study population were patients aged 30 and above who have been diagnosed with any form of dementia (ICD-10 codes F00, F01, F02, F03, G30, G31) in HES between 2 April 2008 and 30 March 2018, which is the study period of interest. Exclusions include patients not residing in England; patients who are a day case, day or night attending (i.e. scheduled) patients, maternity or unknown admissions; those whose care is not provided by an NHS Hospital Trust; those whose discharge and admission dates are invalid, and patients whose data could not be linked to the other datasets. The dataset was aggregated to the GP practice level: the variables which were at the patient level were summarised, such as the mean age, the percentage of female patients, and the total occurrence of hospital admissions.

### Outcomes and covariates

The admissions data were split into two datasets, one for ACSC admissions and one for non-ACSC admissions, based on the primary diagnosis codes for the hospital admission. Individuals could have more than one admission and have both an ACSC and a non-ACSC admission. ACSC admissions are defined as conditions for which appropriate interventions in primary or social care could reduce the need for unplanned hospital admissions [21]. Purdy et al. [18] identified and ICD10 coded ACSCs in the literature and through discussions with expert clinicians, policymakers, and researchers in England. We followed this classification for our study. Additional file 1 lists the ACSC and non-ACSC conditions used in this study. The number of admissions for PWD were related to the potential population at risk by using the number of patients in the practice with dementia from the QOF database [31].

Practice level variables that could influence number of ACSC admissions were (a) mean age and gender of practice patients, (b) deprivation profile of practice (less affluent populations may be less likely to care for patients at home) (c) practice accessibility, measured by two separate variables - whether the practice was in an urban area, and a self-reported patient rating that access to practice was “good” (taken from GP Patient Survey [30]), (d) practice quality score dichotomised as whether or not a practice has a Quality outcomes framework score of >90% achievement versus ≤90%, (e) Practice size (as larger practices might have a partner with a special interest in dementia care and/or that patient management would be more likely to be shared between partners) and, (f) Resources as measured by the percentage of CCG budget which was allocated to mental health. In addition we considered two other variables at the practice level (Region, proportion of patients residing not at home) as potential confounders that might influence both admission rates and GP management due to structural factors such as number of care homes, and hospital bed availability. The data were aggregated to the GP practice level. The data linkage was performed by using the unique GP practice identifiers that are used by the NHS. Two of the authors linked the data independently, and the datasets produced were identical, confirming the linkage.

### Statistical analysis

The outcome variable was the rate of ACSC or non-ACSC admissions divided by the number of patients with dementia multiplied by 100 which was then log transformed [see Eq. 1]

1 $$ \begin{aligned} \text { rate }=\ln \left(\frac{\text { admissions }}{\text { patients with dementia }} \times 100\right) \end{aligned}  $$

Continuous risk factors were mean-centred. We used Bayesian multilevel linear regression models to determine the association between the practice level variables described above and hospital admissions, and to account for the clustering of practices within CCGs [33]. Multilevel models were used as those practices within a given CCG are likely to be more similar to each other than those in other CCGs, producing more valid standard errors [34]. We produced 95% credible intervals, similar to confidence intervals [35]. We examined the intraclass correlation coefficient (ICC), interpreted as the variability attributable to the CCG or practice level, in multivariable models [34]. Step-wise model building was done, starting with a null model, separately for ACSC or non-ACSC admissions, resulting in 24 models (12x2). The intermediate models can be viewed in further detail in Additional file 1. The deviance information criterion (DIC), which penalises for model complexity, was used to assess model fit [34]. We used the *runmlwin* package [33] in Stata 15.1 to run all models.

We hypothesised, a priori, that if a variable was causally related to avoidable hospital admissions, we should see a stronger or differential effect on ACSC compared to non-ACSC admission rates whilst if it reflected population or service contextual factors then it would predict equally for both types of admissions.

## Results

The dataset covered 212 CCGs. While CCGs did merge across the study period, we analysed them as they were recorded at the time point in the dataset. For the ACSC dataset, there were 7,076 practices (range: 31-921 practices per CCG; mean: 289.9 practices per CCG). The ACSC dataset contained 893,224 patient episodes from 512,439 unique patients. The non-ACSC dataset contained 7,063 practices (range: 31-925 practices per CCG; mean: 293.8 practices per CCG) with 705,141 patient episodes, representing 473,803 unique patients. Between the datasets, 7,052 practices overlap, with 24 practices in the ACSC dataset not in the non-ACSC dataset, and 11 practices in non-ACSC dataset not in the ACSC one.

Table 2 shows the distribution of the practice level variables by ACSC and non-ACSC admissions. The proportion of CCG budget allocated to mental health is slightly higher for practices with non-ACSC admissions. ACSC and non-ACSC admissions were similar in all other covariates.

**Table 2:** Descriptive statistics of ACSC and non-ACSC general practices

	ACSC		Non-ACSC	
Continuous Variables	Mean	SD	Mean	SD
Number of admissions from the practice	11.7	10.7	9.3	8.3
Estimated number of patients with dementia in the practice	58.8	42.8	59.4	42.8
Rate of admissions per 100 persons per year (outcome)	7.5	0.8	7.2	0.8
Mean age of patients in the GP practice	39.9	4.3	39.9	4.3
Percent of female patients	50	1.9	50.1	1.9
Mean IMD score of the GP practice	23.4	11.8	23.3	11.8
Percent of patients rating the access to surgery as “good”	78.2	10.6	78.2	10.6
Percent of total CCG budget allocated to Mental Health	11.9	2.2	12.4	2.2
**Categorical Variables**	**Freq**	**Perc**	**Freq**	**Perc**
GP Practice QOF Achievement				
90% or lower	5,120	8.09	4,980	8.00
Above 90%	58,190	91.91	57,252	92.00
GP Practice Population Quartiles				
1 (below 6,527)	28,497	44.97	27,660	44.41
2 (6,528-9,446)	15,424	24.34	15,280	24.53
3 (9,447-12,652)	11,656	18.39	11,592	18.61
4 (above 12,653)	7,791	12.29	7,758	12.45
Admission from home				
No	1,292	2.04	1,537	2.47
Yes	62,076	97.96	60,753	97.53
Rurality				
Urban	54,004	85.22	53,018	85.11
Rural	9,364	14.78	9,272	14.89
Region				
North East	3,428	5.41	3,403	5.46
North West	9,890	15.61	9,777	15.70
Yorkshire & Humber	6,456	10.19	6,325	10.15
East Midlands	5,328	8.41	5,266	8.45
West Midlands	7,469	11.79	7,276	11.68
East of England	6,504	10.26	6,460	10.37
London	10,735	16.94	10,349	16.61
South East	8,468	13.36	8,391	13.47
South West	5,090	8.03	5,043	8.10

**Fig. 1:**
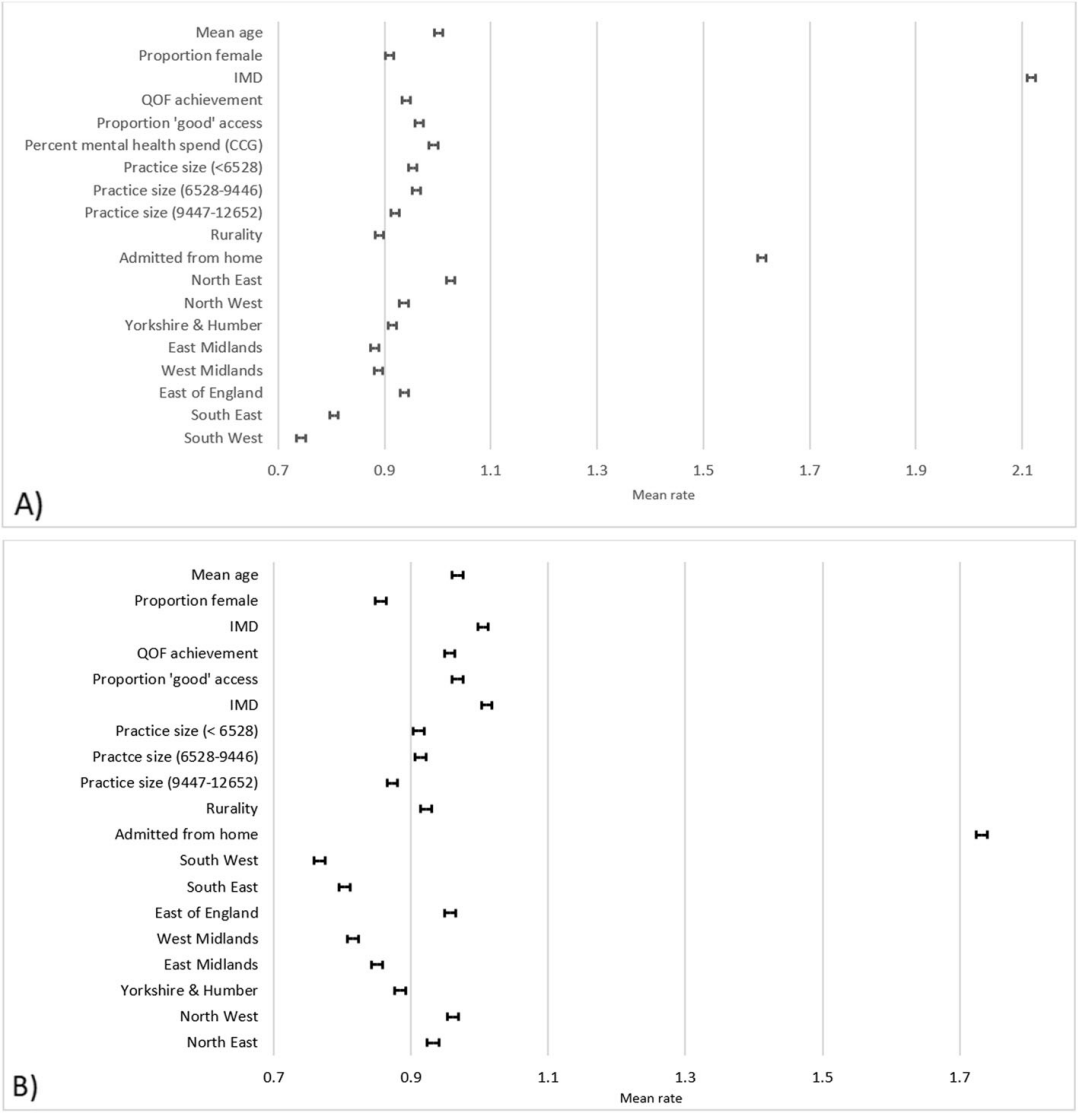
Final model adjusted mean rates with 95% credible intervals, excluding the intercept and admissions year for A) ACSC admissions B) non-ACSC admissions

Table 3 shows the full models (all predictors) for both ACSC and non-ACSC admissions, with the coefficients presented as mean rates, back-transformed from the log rates with exponentiation. The tables in Additional file 2 shows all of the intermediate models. Figure 1 shows the full model, untransformed without intercept and the admission year variable, as the effect sizes were so relatively large as to distort the graph, with 95% credible intervals. The rates relate specifically to GP practices in CCGs, the multilevel structure used for the models. The intercept therefore represents the grand mean GP practice across all CCGs.

**Table 3:** The association of general practice factors with ACSC and non-ACSC hospital admissions

	ACSC			Non-ACSC		
Variable	Mean	95% CI		Mean	95% CI	
Intercept	2115.41	2115.40	2115.41	1764.84	1764.83	1764.85
Practice population mean age per 10 years*	1.00	0.99	1.01	0.97	0.96	0.98
Practice population percent female per 10%*	0.91	0.90	0.92	0.86	0.85	0.86
Practice IMD per 10 points *	1.08	1.07	1.09	1.05	1.05	1.06
Practice QOF Achievement (ref ≤90%)	0.94	0.93	0.95	0.96	0.95	0.96
Percent of practice population rating good access per 10% *	0.96	0.96	0.97	0.97	0.96	0.98
Percent of CCG budget allotted to mental health per 5% *	0.99	0.98	1.00	1.01	1.00	1.02
Practice Population Quartiles						
1 (below 6,527)	0.95	0.95	0.96	0.91	0.90	0.92
2 (6,528-9,446)	0.96	0.95	0.97	0.91	0.91	0.92
3 (9,447-12,652)	0.92	0.91	0.93	0.87	0.86	0.88
4 (above 12,653)	Reference					
Admission Year						
2008	0.17	0.16	0.18	0.18	0.17	0.19
2009	0.35	0.35	0.36	0.34	0.34	0.35
2010	0.46	0.46	0.47	0.43	0.42	0.44
2011	0.54	0.54	0.55	0.51	0.50	0.52
2012	0.66	0.65	0.67	0.59	0.59	0.60
2013	0.73	0.72	0.74	0.64	0.63	0.65
2014	0.82	0.81	0.83	0.71	0.70	0.72
2015	0.91	0.90	0.92	0.78	0.77	0.78
2016	0.99	0.99	1.00	0.86	0.85	0.87
2017	Reference					
Rurality						
Urban	Reference					
Rural	0.89	0.88	0.90	0.92	0.91	0.93
Home admission						
At home	1.61	1.60	1.62	1.73	1.72	1.74
Not from home	Reference					
Region						
London	Reference					
North East	1.02	1.02	1.03	0.93	0.92	0.94
North West	0.94	0.93	0.94	0.96	0.95	0.97
Yorkshire & Humber	0.91	0.91	0.92	0.88	0.88	0.89
East Midlands	0.88	0.87	0.89	0.85	0.84	0.86
West Midlands	0.89	0.88	0.90	0.82	0.81	0.82
East of England	0.94	0.93	0.95	0.96	0.95	0.97
South East	0.81	0.80	0.81	0.80	0.79	0.81
South West	0.74	0.74	0.75	0.77	0.76	0.77

* mean centred variables. The coefficients represent mean rates (transformed from the log rate).

For ACSC admissions, a higher practice IMD (indicating more deprivation; mean rate 1.08 95% CI: 1.07-1.09), older practice mean age (1.00 95% CI: 0.99-1.01), an increase in the percent of the patients ranking the practice as good (0.96, 95% CI 0.96-0.97), and being admitted from home (1.61, 95% CI: 1.60-1.62) are associated with an increase in the admission rate. A larger practice population, a QOF achievement above 90%, increasing proportion of the CCG budget allotted to mental health and rurality are associated with a slight decrease in the rate of admissions. All of the regions apart from the North East are associated with lower admissions than in London.

Table 4 shows the random effects components of the model. ACSC admissions are more variable between GP practices (Variance: 0.351; SD: 0.002) than CCGs (Variance: 0.034; SD: 0.004) (Table 4). Around 91% of the outcome variance is attributable to differences by practice. The DIC shows that the final model fits better than the previous models with the partial coefficients for the ACSC admissions.

**Table 4:** Variability of rates of admission by GP practice and Clinical Commissioning Group (CCG)

	ACSC				Non-ACSC			
Variable	Null model		Full model		Null model		Full model	
**Variance**	Var	SD	Var	SD	Var	SD	Var	SD
Practice level	0.618	0.003	0.351	0.002	0.573	0.003	0.341	0.002
CCG level	0.067	0.007	0.034	0.004	0.053	0.005	0.024	0.003
**Intra-class correlation coefficient**								
Practice ICC	0.902		0.910		0.915		0.934	
CCG ICC	0.098		0.090		0.085		0.066	
**Model DIC**	149553.57		112254.30		142303.69		112254.30	

For non-ACSC admissions, the overall patterning of the predictor variables was broadly similar to that for ACSC admissions, with a few exceptions. A lower practice mean age is associated with a decrease in non-ACSC admissions, while the proportion of the CCG budget allotted to mental health is associated with an increase in admissions. All other regions have lower admissions rates for non-ACSC admissions compared to London. Non-ACSC admissions are more variable between GP practices (Variance: 0.341; SD: 0.002) than CCGs (Variance: 0.024; SD: 0.003) (Table 4). Around 93% of the outcome variance is attributable to differences by practice.

## Discussion

We found a range of practice level predictors for hospital admissions. For example, higher deprivation and admissions from home are associated with an increase in the admission rate for both ACSC and Non-ACSC admissions. A larger practice population, a QOF achievement above 90% and rurality are associated with a slight decrease in the rate of admissions, regardless of the reason for admission. Whilst it is tempting to think that better practice quality is causally related, the observation that this predicts lower admission rates for both ACSC and non-ACSC admissions suggests that this is non-causal and is possibly subject to unmeasured confounding. It is possible that better quality practices could have better outcomes for both types of admissions. Practice mean age and the proportion of the CCG budget allotted to mental health show small but divergent effects for ACSC and non-ACSC admissions, indicating that they could plausibly be causally related to avoidable admissions. More than 90% of the variability in hospital admissions of both types are attributable to GP practice level differences.

Practice mean age and the proportion of the CCG budget allotted to mental health show small but divergent effects for ACSC and non-ACSC admissions, indicating that they could plausibly be causally related to avoidable admissions. More than 90% of the variability in hospital admissions of both types are attributable to GP practice level differences. Practices which had an older mean age tended to have higher admission rates in ACSC versus non-ACSC admissions, counter to our a priori hypothesis. This may reflect the greater burden of dementia in these populations so that support and social care services are overwhelmed, though this is a post-hoc explanation. An increase in the percent of the CCG budget allocated to mental health care was associated with a reduction in ACSC admissions but an increase in non-ACSC admissions. It is not clear to us if this is causal, as much of this budget relates to psychiatric morbidity, but this requires further investigation. If this association is real then it may actually result in longer term cost savings, assuming a reasonable amount of the budget is used to provide support for carers and community care.

Our Results are comparable to previous studies. Practice factors, as suggested by Busby et al. [12] were linked to admissions in general, but many of the effect sizes in our study, such as for practice mean age, were very small. One of Busby et al’s [12] primary findings was that deprivation, in their case 90th centile of the IMD, was linked to 16% (CI 14-18%) higher ACSC admissions, a finding replicated here. However, our study found that rurality was not linked to increased rates of ACSC or non-ACSC admissions, in contrast to work by Thorpe et al. [36], which found that those living in more rural areas had significantly more ACSC hospitalisations than those in urban ones. This suggests that the rural English context is different to the American one; perhaps hospital referrals are more encouraged in rural America.

The strengths of our study are the use of robust, appropriate methodology, and the completeness of the HES dataset. This means that we have captured the majority of hospital admissions for PWD. Further, this study specifically examines PWD, a group known to be complex in needs, something not done before for ACSCs. The HES data do not include private hospital admissions though these are predominantly elective procedures and most hospitals in the UK are public [37]. Further, we found no evidence of an effect of hospital type, i.e. private or public, when examining quality of care and controlling for case-mix and patient characteristics [37].

There are several important limitations; our denominator data come from the QOF register and is likely to be incomplete and worse for more disorganised practices. This will result in a biased over-estimation of the rate due to an underestimation of the dementia population. As Sommerlad et al. [38], in their study of patients in South London comparing patients” diagnoses in a memory clinic versus HES records, note that the sensitivity for an individual nonelective admission is 63.3% and the specificity is 96.6%. This means that our practice data may be missing some PWD before aggregation. Further, people from ethnic minority groups have more missed diagnoses [38], and therefore our sample, pre-aggregation, may have some systematic biases which could carry through to the practice level data. Our data on practice quality is general in nature and it is possible that the correlation between this measure and prompt preventative care is not that strong. There are other practice factors that we did not have such as whether the practice was a teaching practice, the age of the partners and whether any partner had a special interest in elderly and/or dementia patients. We could not include local authority data, due to the complexity of the multiple-membership models required, which did not converge. For similar reasons, we could not include community healthcare trusts. The percent of the CCG budget allocated to mental health may not necessarily translate to a sufficiently large spend on PWD, though it is included explicitly in this allocation [39]. We also did not explore NHS Provider characteristics which might have had an impact on admissions and presumably explain some of the wide regional variations.

## Conclusions

In conclusion, we have examined a variety of factors that could explain avoidable admissions for PWD and found most were equally predictive for avoidable and non-avoidable admissions. However, there were some unexplained geographical differences, particularly in relation to the percent of the CCG budget allocated to mental health, which needs further exploration to see if this is actually spent in such a way as to benefit PWD. The NHS has emphasised place-based commissioning as a key element of its Long Term Plan, which may account for some of these geographic differences [6]. Furthermore, an independent dementia care assessment commissioned by the NHS in 2018 found a high amount of variability in CCGs, with many rated as inadequate or requiring improvement, and performance has not improved year on year [40]. Further, the high amount of variation found at the practice level, even accounting for practice characteristics, implies that practices and other services in the admission pathway may be applying guidance inconsistently, or that guidance and local services for referring and treating comorbidities for PWD vary and have potential scope for improvement. Furthermore, as the differences between practices may also include the differences between individuals in those practices, the influence of these individuals should be examined in future work.

## Supplementary Information

**Additional file 1** This additional file shows which conditions were considered ACSCs and which were considered non-ACSCs

**Additional file 2** This additional file shows the intermediate models between the null and final model, untransformed

## Data Availability

The Hospital Episode Statistics (HES) data that support the findings of this study are available from NHS Digital but restrictions apply to the availability of these data, which were used under license for the current study, and so are not publicly available. The other datasets supporting the conclusions of this article are available from NHS Digital and Public Health England: ∙ The Quality Outcomes Framework: https://digital.nhs.uk/data-and-information/data-tools-and-services/data-services/general-practice-data-hub/quality-outcomes-framework-qof ∙ CCG Programme Budgeting Benchmarking Tool: https://www.england.nhs.uk/prog-budgeting/ ∙ GP Patient Survey: https://www.england.nhs.uk/statistics/statistical-work-areas/gp-patient-survey/ ∙ GP Practice Profiles: https://fingertips.phe.org.uk/profile/general-practice

## Abbreviations

ACSC: Ambulatory Care Sensitive Condition
CCG: Clinical Commissioning Group
CI: Credible Interval
DIC: Deviance Information Criterion
GP: General Practice
HES: Hospital Episode Statistics
ICC: Intraclass Correlation
IMD: Index of Multiple Deprivation
NHS: National Health Service
PWD: People with Dementia
QOF: Quality Outcomes Framework

## References

[1] Public Health England (2020). Dementia profile.

[2] T P, C B. Dementia: policy, services, and statistics. Technical report. House of Commons, London. 2019.

[3] for Health and Care Excellence NI. NICEimpact: dementia. Technical report. NICE, London. 2020.

[4] Public Health England (2018). Dementia: Applying All Our Health.

[5] England N. Five Year Forward View. Technical report. NHS, London. 2014.

[6] England N. The NHS Long Term Plan. Technical report. NHS, London. 2019.

[7] Public Health England (2015). Reasons why people with dementia are admitted to a general hospital in an emergency.

[8] EL S, MR B, L J (2009). Dementia in the acute hospital: prospective cohort study of prevalence and mortality. British J Psych.

[9] S T, M D, A A (2013). Causes of hospital admission for people with dementia: a systematic review and meta-analysis. J Am med Dir Assoc.

[10] P K, AR M, MK G (2015). The influence of primary care quality on hospital admissions for people with dementia in England: a regression analysis. Plos one.

[11] Wolf D, Rhein C, Geschke K, Fellgiebel A (2018). Preventable hospitalizations among older patients with cognitive impairments and dementia. Int Psychogeriatr.

[12] J B, S P, W H (2017). How do population, general practice and hospital factors influence ambulatory care sensitive admissions: a cross sectional study. BMC Family Practice.

[13] L L. Counting the cost: Caring for people with dementia on hospital wards. Technical report. Alzheimer’s Society, London. 2009.

[14] Shepherd H, Livingston G, Chan J, Sommerlad A. Hospitalisation rates and predictors in people with dementia: a systematic review and meta-analysis. BMC Med. 2019; 17(130). 10.1186/s12916-019-1369-7 PMC6628507 31303173

[15] Tropea J, LoGiudice D, Liew D, Gorelik A, Brand C (2016). Poorer outcomes and greater healthcare costs for hospitalised older people with dementia and delirium: a retrospective cohort study. Int J Geriatr Psychiatr.

[16] C E, M P, J B (2013). Homeward bound or bound for a home? Assessing the capacity of dementia patients to make decisions about hospital discharge: comparing practice with legal standards. Int J Law Psych.

[17] Lin PJ, Zhong Y, Fillit HM, Cohen JT, Neumann PJ (2017). Hospitalizations for ambulatory care sensitive conditions and unplanned readmissions among Medicare beneficiaries with Alzheimer’s disease. Alzheimer’s and Dementia.

[18] S P, T G, C S (2009). Ambulatory care sensitive conditions: terminology and disease coding need to be more specific to aid policy makers and clinicians. Pub Health.

[19] Sundmacher L, Fischbach D, Schuettig W, Naumann C, Augustin U, Faisst C (2015). Which hospitalisations are ambulatory care-sensitive, to what degree, and how could the rates be reduced? Results of a group consensus study in Germany. Health Policy.

[20] J B, S P, W H (2017). Opportunities for primary care to reduce hospital admissions: a cross-sectional study of geographical variation. Br J Gen Pract.

[21] Elect N. Directory of Ambulatory Emergency Care for Adults. Technical report. NHS, London. 2016.

[22] NHS Digital (2021). Ambulatory Care Sensitive Conditions (ACSC).

[23] Pimouguet C, Rizzuot D, Fastbom J, Lagergren M, Fratiglioni L, Xu W (2016). Influence of Incipient Dementia on Hospitalization for Primary Care Sensitive Conditions: A Population-Based Cohort Study. J Alzheimer’s Disease.

[24] Phelan EA, Borson S, Grothaus L, Balch S, Larson EB (2012). Association Between Incident Dementia and Risk of Hospitalization. JAMA.

[25] JL G, MD S, KS G, SL M (2012). Hospital Transfers of Nursing Home Residents with Advanced Dementia. J Am Ger Soc.

[26] van Loenen T, van den Berg MJ, Westert GP, Faber MJ (2014). Organizational aspects of primary care related to avoidable hospitalization: a systematic review. Fam Pract.

[27] NHS Clinical Commissioners (NHSCC) (2019). About CCGs.

[28] Digital N. Hospital Episode Statistics (HES).

[29] Public Health England (2018). General practice profiles.

[30] NHS England (2018). GP patient survey.

[31] NHS Digital (2018). Quality Outcomes Framework (QOF).

[32] NHS England (2018). Programme Budgeting.

[33] G L, C C (2013). runmlwin - A Program to Run the MLwiN Multilevel Modelling Software from within Stata. J Stat Software.

[34] A G, JB C, HS S (2014). Bayesian Data Analysis.

[35] Browne WJ (2019). MCMC Estimation in MLwiN, v3.03.

[36] Thorpe JM, van Houtven C, Sleath BL, Thorpe CT. Rural-urban differences in preventable hospitalizations among community-dwelling veterans with dementia. J Rural Health. 2010; 26(2). 10.1111/j.1748-0361.2010.00276.x PMC3691102 20447001

[37] Moscelli G, Gravelle H, Siciliani L, et al. The effect of hospital ownership on quality of care: evidence from England. Technical report. Centre for Health Economics, York. 2017.

[38] Sommerlad A, Perera G, Singh-Manoux A, Lewis G, Stewart R, Livingston G (2018). Accuracy of general hospital dementia diagnoses in England: Sensitivity, specificity, and predictors of diagnostic accuracy 2008-2016. Alzheimer’s and Dementia.

[39] NHS. NHS Mental Health Dashboard. 2021. https://www.england.nhs.uk/mental-health/taskforce/imp/mh-dashboard/. Date accessed: 01 Feb 2021.

[40] Hughes J, England N. CCG Dementia Assessment 2017/18: Independent Panel Commentary. Technical report. NHS, London. 2018.

