# Supplementary material for: Associations of GP practice characteristics with the rate of ambulatory care sensitive conditions in people living with dementia in England: an ecological analysis of routine data
Source: BMC Health Serv Res. 2021 Jun 29;21:613. doi: 10.1186/s12913-021-06634-7 (PMC8240405; doi:10.1186/s12913-021-06634-7)
Supplement: Supplementary file 1 — Additional file 1 This additional file shows which conditions were considered ACSCs and which were considered non-ACSCs [file 12913_2021_6634_MOESM1_ESM.pdf]

Additional file 1: ACSC and non-ACSC conditions

ACSC conditions	Non-ACSC conditions
deep vein thrombosis	other infectious and parasitic disease
pulmonary embolism	neoplasms
pneumothorax	other endocrine/nutritional/metabolic disorder
pleural effusions	other mental/behavioural disorders
asthma	other nervous system diseases
COPD	other diseases of the eye and adnexa
community acquired pneumonia	ischaemic heart diseases
LRTI without COPD	other respiratory tract infections
congestive heart failure	other digestive system diseases
tachycardias	other diseases of the skin
low risk chest pain	muscukoskeletal diseases
Transient ischaemic attack	other genitourinary diseases
stroke	pregnancy/childbirth related diseases
seizure	abnormal clinical and lab findings
acute headache	injury/external causes of morbidity/mortality
upper GI haemorrhage	factors influencing health/health services
lower GI haemorrhage	
gastroenteritis	
abnormal liver function	
anaemia	
diabetes	
cellulitis of limb	
known oesophageal stenosis	
PEG related complications	
self-harm & accidental overdose	
falls	
UTI	
acutely hot painful joint	
appendicular fractures	
non-traumatic vertebral fractures	
low risk pubic rami fractures	
hip pain secondary to fall	
acute abdominal pain	
abscess requiring drainage	
head injury	
acute painful bladder outflow obstructi	
renal/uteric stones	
gross haematuria	
chronic indwelling catheter related problems	
acute scrotal pain	
