# Supplementary material for: Associations of GP practice characteristics with the rate of ambulatory care sensitive conditions in people living with dementia in England: an ecological analysis of routine data
Source: BMC Health Serv Res. 2021 Jun 29;21:613. doi: 10.1186/s12913-021-06634-7 (PMC8240405; doi:10.1186/s12913-021-06634-7)
Supplement: Supplementary file 2 — Additional file 2 This additional file shows the intermediate models between the null and final model, untransformed [file 12913_2021_6634_MOESM2_ESM.pdf]

Additional file 2: Intermediate models, estimates untransformed

	ACSC			Non-ACSC		
	Mean	95% CI		Mean	95% CI	
**Model 1**						
Intercept	1687.31	1687.3	1687.32	1359.61	1359.6	1359.62
Practice population mean age *	0.83	0.82	0.83	0.84	0.83	0.84
	ACSC			Non-ACSC		
	Mean	95% CI		Mean OR	95% CI	
**Model 2**						
Intercept	1695.79	1695.78	1695.8	1365.85	1365.84	1365.85
Practice population mean age *	0.85	0.84	0.85	0.86	0.85	0.87
Practice population percent female *	0.78	0.77	0.79	0.74	0.73	0.74
	ACSC			Non-ACSC		
	Mean	95% CI		Mean	95% CI	
**Model 3**						
Intercept	1701.77	1701.76	1701.77	1369.06	1369.05	1369.06
Practice population mean age *	0.92	0.92	0.93	0.91	0.91	0.92
Practice population percent female *	0.84	0.83	0.85	0.78	0.77	0.78
Practice IMD *	1.01	1	1.02	1.01	1	1.01
	ACSC			Non-ACSC		
	Mean	95% CI		Mean	95% CI	
**Model 4**						
Intercept	1911.76	1911.75	1911.77	1499.12	1499.11	1499.13
Practice population mean age *	0.92	0.91	0.93	0.91	0.91	0.92
Practice population percent female *	0.85	0.84	0.86	0.79	0.78	0.79
Practice IMD *	1.01	1	1.02	1.01	1	1.01
Practice QOF Achievement (ref ≤90%)	0.88	0.87	0.89	0.91	0.9	0.91
	ACSC			Non-ACSC		
	Mean	95% CI		Mean	95% CI	
**Model 5**						
Intercept	1874.27	1874.27	1874.28	1469.37	1469.36	1469.38
Practice population mean age *	0.96	0.96	0.97	0.95	0.94	0.96
Practice population percent female *	0.84	0.83	0.85	0.77	0.76	0.78
Practice IMD *	1.01	1	1.01	1	1	1.01
Practice QOF Achievement (ref ≤90%)	0.9	0.89	0.9	0.92	0.91	0.93
Percent of practice population rating good access *	0.87	0.86	0.87	0.88	0.87	0.89
	ACSC			Non-ACSC		
	Mean	95% CI		Mean	95% CI	
**Model 6**						
Intercept	1877.21	1877.2	1877.21	1473.32	1473.31	1473.33

Practice population mean age *	0.97	0.96	0.97	0.96	0.95	0.96
Practice population percent female *	0.84	0.84	0.85	0.77	0.77	0.78
Practice IMD *	1.01	1	1.01	1	1	1.01
Practice QOF Achievement (ref ≤90%)	0.9	0.89	0.91	0.93	0.92	0.93
Percent of practice population rating good access *	0.87	0.86	0.88	0.88	0.88	0.89
Percent of CCG budget allotted to mental health *	1.3	1.3	1.31	1.3	1.3	1.31
	ACSC			Non-ACSC		
	Mean	95% CI		Mean	95% CI	
**Model 7**						
Intercept	1658.73	1658.72	1658.74	1268.34	1268.34	1268.35
Practice population mean age *	0.95	0.94	0.96	0.94	0.93	0.94
Practice population percent female *	0.91	0.9	0.92	0.85	0.85	0.86
Practice IMD *	1.01	1	1.01	1	1	1.01
Practice QOF Achievement (ref ≤90%)	0.92	0.91	0.92	0.95	0.94	0.96
Percent of practice population rating good access *	0.86	0.85	0.87	0.87	0.86	0.88
Percent of CCG budget allotted to mental health *	1.3	1.29	1.31	1.29	1.29	1.3
Practice Population Quartiles						
1 (below 6,527)	1.2	1.19	1.21	1.26	1.25	1.27
2 (6,528-9,446)	1.07	1.07	1.08	1.08	1.07	1.09
3 (9,447-12,652)	1.06	1.05	1.07	1.07	1.06	1.08
4 (above 12,653)	Reference					
	ACSC			Non-ACSC		
	Mean	95% CI		Mean	95% CI	
**Model 8**						
Intercept	2807	2806.99	2807	2365.29	2365.28	2365.3
Practice population mean age *	0.98	0.98	0.99	0.96	0.95	0.97
Practice population percent female *	0.93	0.92	0.93	0.87	0.86	0.88
Practice IMD *	1.01	1	1.02	1.01	1	1.01
Practice QOF Achievement (ref ≤90%)	0.94	0.93	0.95	0.96	0.95	0.96
Percent of practice population rating good access *	0.96	0.95	0.97	0.97	0.96	0.97
Percent of CCG budget allotted to mental health *	0.99	0.98	1	1.01	1	1.02
Practice Population Quartiles						
1 (below 6,527)	1.06	1.05	1.07	1.12	1.11	1.12
2 (6,528-9,446)	1.02	1.01	1.03	1.03	1.02	1.04
3 (9,447-12,652)	1.04	1.03	1.04	1.04	1.03	1.05
4 (above 12,653)	Reference					

Admission Year						
2008	0.17	0.16	0.18	0.17	0.16	0.18
2009	0.35	0.34	0.36	0.34	0.33	0.35
2010	0.46	0.45	0.47	0.43	0.42	0.44
2011	0.54	0.54	0.55	0.51	0.5	0.51
2012	0.66	0.65	0.67	0.59	0.58	0.6
2013	0.73	0.72	0.74	0.64	0.63	0.65
2014	0.82	0.82	0.83	0.71	0.7	0.71
2015	0.91	0.9	0.92	0.77	0.77	0.78
2016	0.99	0.98	1	0.86	0.85	0.87
2017	Reference					

	ACSC			NON-ACSC		
	Mean	95% CI		Mean	95% CI	
**Model 9**						
Intercept	2812.11	2812.1	2812.12	2369.14	2369.13	2369.15
Practice population mean age *	1.01	1	1.01	-0.98	-0.97	-0.98
Practice population percent female *	0.92	-0.92	-0.93	-0.87	-0.86	-0.88
Practice IMD *	1.01	1	-1.02	1.01	1	1.01
Practice QOF Achievement (ref ≤90%)	0.94	-0.93	-0.95	-0.96	-0.95	-0.96
Percent of practice population rating good access *	0.96	-0.96	-0.97	-0.97	-0.96	-0.98
Percent of CCG budget allotted to mental health *	0.99	-0.98	-1	1.01	1	1.02
Practice Population Quartiles						
1 (below 6,527)	1.08	1.07	1.09	1.13	1.12	1.14
2 (6,528-9,446)	1.03	1.03	1.04	1.04	1.03	1.05
3 (9,447-12,652)	1.04	1.03	1.05	1.05	1.04	1.05
4 (above 12,653)	Reference					
Admission Year						
2008	0.17	0.16	0.18	0.17	0.16	0.18
2009	0.35	0.34	0.36	0.34	0.33	0.35
2010	0.46	0.45	0.47	0.43	0.42	0.44
2011	0.54	0.53	0.55	0.51	0.5	0.51
2012	0.66	0.65	0.67	0.59	0.58	0.6
2013	0.73	0.72	0.74	0.64	0.63	0.65
2014	0.82	0.82	0.83	0.71	0.7	0.71
2015	0.91	0.9	0.92	0.77	0.77	0.78
2016	0.99	0.98	1	0.86	0.85	0.87
2017	Reference					
Rurality						
Urban	Reference					
Rural	0.89	0.88	0.9	0.92	0.91	0.93

	ACSC			NON-ACSC		
	Mean	95% CI		Mean	95% CI	
**Model 10**						
Intercept	1749.56	1749.56	1749.57	1365.34	1365.33	1365.35
Practice population mean age *	1	0.99	1.01	0.97	0.96	0.97
Practice population percent female *	0.91	0.9	0.92	0.86	0.85	0.86
Practice IMD *	1.01	1	1.02	1.01	1	1.01
Practice QOF Achievement (ref ≤90%)	0.94	0.93	0.95	0.96	0.95	0.96

Percent of practice population rating good access *	0.96	0.96	0.97	0.97	0.96	0.98
Percent of CCG budget allotted to mental health *	1	0.99	-1	1.01	1.01	1.02
Practice Population Quartiles						
1 (below 6,527)	1.09	1.08	1.1	1.15	1.14	1.16
2 (6,528-9,446)	1.04	1.03	1.05	1.05	1.04	1.05
3 (9,447-12,652)	1.04	1.04	1.05	1.05	1.04	1.06
4 (above 12,653)	Reference					
Admission Year						
2008	0.17	0.16	0.18	0.18	0.17	0.19
2009	0.35	0.35	0.36	0.34	0.34	0.35
2010	0.46	0.46	0.47	0.43	0.42	0.44
2011	0.54	0.54	0.55	0.51	0.5	0.52
2012	0.66	0.65	0.67	0.59	0.59	0.6
2013	0.73	0.72	0.74	0.64	0.63	0.65
2014	0.82	0.81	0.83	0.71	0.7	0.72
2015	0.91	0.9	0.92	0.78	0.77	0.78
2016	0.99	0.99	1	0.86	0.85	0.87
2017	Reference					
Rurality						
Urban	Reference					
Rural	0.89	0.88	0.9	0.92	0.91	0.93
Home admission						
At home	1.61	1.6	1.62	1.73	1.73	1.74
Not from home	Reference					
